# Correlation between caries activity and salivary microbiota in preschool children

**DOI:** 10.3389/fcimb.2023.1141474

**Published:** 2023-04-11

**Authors:** Xiuyan Lin, Yuan Wang, Zhe Ma, Meng Xie, Zhuo Liu, Jinghui Cheng, Yuzhao Tian, Hong Shi

**Affiliations:** ^1^ Department of Pediatric Dentistry, Hospital of Stomatology and Hebei Provincial Key Laboratory of Stomatology, Hebei Medical University, Shijiazhuang, China; ^2^ Department of Stomatology, Zhao County Maternal and Child Health Hospital, Shijiazhuang, China; ^3^ Department of Preventive Dentistry, Hospital of Stomatology and Hebei Provincial Key Laboratory of Stomatology, Hebei Medical University, Shijiazhuang, China

**Keywords:** caries activity, microbial community, early childhood caries, 16S rRNA gene, saliva, preschool children

## Abstract

Early childhood caries (ECC) is the most common chronic infectious oral disease in preschool children worldwide. It is closely related to the caries activity (CA) of children. However, the distribution characteristics of oral saliva microbiomes in children with different CA are largely underexplored. The aim of this study was to investigate the microbial community in saliva of preschool children with different CA and caries status, and to analyze the difference of microbial community in saliva of children with different CA and its correlation with ECC. Subjects were divided into 3 groups based on the Cariostat caries activity test: Group H, high CA (n=30); Group M, medium CA (n = 30); Group L, low CA (n=30). Questionnaire survey was used to explore the related influencing factors of CA. According to the caries status (on the basis of decayed mising filled teeth), these subjects were divided into caries-free group (dmft=0, n=19), caries-low group (0 < dmft ≤ 4, n=27) and caries-high group (dmft > 4, n=44). Microbial profiles of oral saliva were analyzed using 16S rRNA gene sequencing. There were significant differences in the microbial structure (*P* < 0.05). *Scardovia* and *Selenomonas* were the biomarkers of both H group and high caries group. The genus *Abiotrophia* and *Lautropia* were the biomarkers of both the L group and the low caries group, while the *Lactobacillus* and *Arthrospira* spp. were significantly enriched in the M group. The area under the ROC curve of the combined application of dmft score, age, frequency of sugary beverage intake, and the genus *Scardovia*, *Selenomonas*, and *Campylobacter* in screening children with high CA was 0.842. Moreover, function prediction using the MetaCyc database showed that there were significant differences in 11 metabolic pathways of salivary microbiota among different CA groups. Certain bacteria genera in saliva such as *Scardovia* and *Selenomonas* may be helpful in screening children with high CA.

## 1 Introduction

Early childhood caries (ECC) is a global problem that disturbs children’s physical and mental health. A survey by Uribe et al. in 2021 showed that ECC affected 48% of preschool children worldwide, and its prevalence ranged from 16% (Singapore) to 89% (China), with regional differences in distribution and serious prevalence (Uribe et al., 2021). The China Oral Health Epidemiological Survey Report in 2018 demonstrated that the caries prevalence of children aged 3, 4, and 5 years old was 50.8%, 63.6%, and 71.9%, respectively, while the treatment rates were only 1.5%, 2.9%, and 4.1%, respectively (Wang, 2018). Even with treatment, about 40% of cases will relapse within one year (Berkowitz et al., 2011). Of particular note is the increasing prevalence of ECC among 5-year-old children in China, which is 5.9% higher than a decade ago (Wang, 2018). Severe ECC not only affects children’s growth, development, and quality of life (Lara et al., 2022), but also has a negative impact on their parents and society (Zou et al., 2022). Early prediction and prevention of ECC have been the focus of research worldwide.

ECC is a public health problem caused by the interaction of cariogenic bacteria, dietary habits and some socioeconomic factors (Pierce et al., 2019; Zou et al., 2022). Cariogenic microorganisms present in the oral cavity play a key role in the occurrence and development of ECC, and the formed biofilm can metabolize carbohydrates to produce organic acids, resulting in local pH decline and demineralization of dental hard tissues (Marsh, 1994). Wade et al. (Wade, 2013) found that oral cavity is one of the most important habitats for microorganisms, with about 1000 different microbial species, and it is of great significance to maintain the ecosystem balance between host and microorganisms for maintaining oral health.

In recent years, the understanding of microorganisms and the pathogenesis of ECC has gradually deepened. Li et al. (Li et al., 2018) assessed the oral microbial diversity of 12-to 24-month-old children using a cohort study and found that the colonization, development and stability of the oral microflora developed dynamically during tooth eruption, and the oral microbial community gradually matured and stabilized with the growth and development of the children. Several studies described that there are differences in the oral microbiota composition between children without and with caries (Jiang et al., 2016; Vieira et al., 2019; de Jesus et al., 2021). A 12-month study examined the changes of supragingival microbiota during the occurrence and progression of dental caries in children aged 3 years old, and the results indicated that the microbial changes before caries onset might be used for the early diagnosis and prediction of dental caries (Xu H. et al., 2018). Zhu et al. (Zhu et al., 2018) also believed that observing the status of salivary microbiota had the potential to predict the recurrence of ECC. Tang et al. (Tang et al., 2022) explored and analyzed the association between dental caries and oral microflora at the functional level, and the results showed that the functional pathways involving glycolysis and acid production were significantly upregulated in the high caries group. Dysbiosis of biofilms as well as changes in bacterial composition occur when excessive and frequent intake of carbohydrates leads to acid production that exceeds the buffering capacity of the healthy microbiome (Tanner et al., 2018). Previous studies (Xu L. et al., 2018; Zheng et al., 2018; Grier et al., 2021) compared the oral microbiota composition between children with and without ECC and found that the taxonomic groups such as *Veillonella parvula*, *Rothia mucilaginosa*, *Actinomyces*, *Lactobacillus fermentum*, and *Neisseria sica* were significantly associated with ECC. In addition, the interaction between *Candida albicans* and *Streptococcus mutans* can also promote the occurrence and development of ECC (Yang et al., 2018). However, the microflora found in the mouth of children without caries included *Fusobacterium*, *Kingella*, *Leptotrichia*, *Shuttleworthia*, and *Roseia* (Zhang et al., 2015; Xu L. et al., 2018). Up to now, there have been divergent conclusions on the composition of oral microbiota in children with ECC. The relationship between the changes of oral microbiota and the host as well as the related influencing factors during the development of ECC is still unclear, especially the relationship between the changes of children’s caries activity (CA) and the composition and changes of oral microbiota as well as their roles in the caries process have not been reported yet.

Caries-risk assessment (CRA) is one of the basic factors for the planning of caries prevention and treatment strategies (Caglar et al., 2008), and the caries activity test (CAT) for the screening of susceptible children is one of the most critical indicators (Nishimura et al., 2008). Previous studies have shown that the continuous high level and fluctuation of CAT value are closely related to the occurrence and development of ECC, and maintaining a continuous low level of CA in children is of great significance for the prevention of ECC (Liu et al., 2018). Xuan et al. (Xuan et al., 2017) found that CAT value had a significant positive correlation with the incidence rate of ECC. The higher CAT value in dental plaque of children, the higher the risk for dental caries in children. In particular, CA is closely related to the acidogenic ability, quantity and type of cariogenic microorganisms (Lee et al., 2016) The CA of 406 children was detected by Rodis et al. (Rodis et al., 2009) using Cariostat method, and the results showed that *Streptococcus mutans*, *Streptococcus sobrinus*, S*treptococcus salivarius*, *Lactobacillus casei*, *Lactobacillus plantarum*, and *Lactobacillus fermentum* were all related to CA in children, and the number of bacteria other than *Streptococcus salivarius* was in an approximate linear relationship with CAT value. However, Li et al. (Li et al., 2006) observed that the CAT value of some young children with high decayed missing filled-teeth (dmft) scores was still in the relatively safe range or critical value, while the CAT value of children with low dmft scores was high. Therefore, it is suggested that whether there are some oral flora changes related to the fluctuation of CA in children? What changes in microbiota can trigger the development and progression of ECC? What are the differences in the composition of oral microbial communities among children with different CA? What is the correlation with caries status in children? Could these differences reverse the development of ECC? At present, the above-mentioned related studies are rare, especially on whether the sensitive oral microorganisms causing CA fluctuation in children, children’s diet, health habits and other factors can be applied to find and establish risk diagnosis models and methods targeted for prediction and prevention of ECC, which have not been reported yet.

Meanwhile, different microorganisms colonize different parts of the mouth, including saliva, tongue, gingival crevicular fluid, and buccal mucosa (Shaw et al., 2017; Mark Welch et al., 2019; Zhang et al., 2021). However, the saliva microflora has long-term stability and is an essential reservoir of oral microorganisms, containing all the microorganisms from diverse ecosystems of the oral cavity (Shaw et al., 2017). Moreover, saliva, as an easily available, relatively stable and cheap biological fluid, has long been a biomarker of health and disease (Giannobile et al., 2011; Segata et al., 2012). Therefore, determining the composition of the saliva microflora under different conditions is also crucial for understanding the complete oral microflora and its effects on oral health and diseases (Shaw et al., 2017). In this study, preschool children aged 3–5 years old with different CA were selected as the research subjects, and the composition and structure of their oral saliva microbiota were observed and analyzed using the high-throughput sequencing technology, as well as the related influencing factors of CA, to explore the differences and correlations of saliva microbiota among children with different CA and caries status, so as to provide a scientific basis for exploring the role of salivary microbiota and CA changes in the occurrence of ECC and establishing a comprehensive prediction and diagnosis model based on salivary microbiota, oral health and CA.

## 2 Materials and Methods

### 2.1 Study population

A total of 90 children aged 3–5 years were recruited in 6 kindergartens in zhao county, hebei province, China. Inclusion criteria for all selected subjects were as follows: (1) Children aged 3-5 years (36-60 months) with only primary dentition; (2) No systemic diseases, infectious disease and significant congenital anomalies; (3) No use of antibiotics, probiotics and topical application of fluoride within three months prior to the study; (4) No visually detectable enamel or dentin hypoplasia; (5) No orthodontic appliances or accessories. This study was approved by the Ethics Committee of Dental Hospital, Hebei Medical University, China (No. [2018] 028). Written informed consent was obtained from all parents or legal guardians of eligible participating children.

### 2.2 Cariostat test, questionnaire survey and oral examination

#### 2.2.1 Cariostat test and questionnaire survey

In accordance with the instructions of the Cariostat kit (GangDa Medical Technology Co. LTD., Beijing, China), the examiners used the sterile cotton swab to scrub the buccal surfaces of the maxillary molars and the labial surfaces of mandibular incisors 3–5 times with a gentle movement. The cotton swab was immersed into the culture medium ampule. The sample was incubated at 37°C for 48 hours. The colour of the culture medium was compared with the standard colour chart provided by the manufacturer under natural light and the scores ranging from 0 to 3 were evaluated by reference. In this study, a modified scale was used, where the interval between 0–1.0, 1.0–2.0, and 2.0–3.0 were divided into halves. Values lies in 0-1.0 for low caries risk children were defined as Group L (n=30), 1.5 for medium caries risk children were defined as Group M (n=30), 2.0-3.0 for high caries risk children were defined as Group H (n=30).

A structured questionnaire was used to obtain the data on sociodemographic characteristics of children and parents/guardians (name, gender, age, mode of mother’s delivery), dietary habits of daily life (mode of feeding within 6 months after birth, frequency of sugary beverages consumption, frequency of snacks or sweets consumption), and oral health care behaviors (using fluoride toothpaste, frequency and time of tooth brushing). The questionnaire was completed by investigators conducting one-on-one on-site questioning of the child’s parents/caregivers at the designated time and location.

#### 2.2.2 Oral examination

According to the the 5th edition of Basic Methods of Oral Health Survey of WHO (World Health Organization (WHO), 2013), 90 children were examined by two experienced pediatric dentists under natural light using a disposable dental mirror and a community periodontal index (CPI) probe, and the dmft scores were recorded to assess the caries status of the deciduous dentition. Caries experience level was assessed based on age and dmft scores (Vieira et al., 2008). Two dmft cut-off values (0 < dmft ≤ 4 and dmft > 4) were applied. The subjects were divided into caries free (CF) group (dmft=0, n=19), low-caries (LC) group (0 < dmft ≤ 4, n=27), and high-caries (HC) group (dmft > 4, n=44) on the basis of the results of the clinical examination. No x-ray was taken. Before the study, two oral examiners were trained in theoretical knowledge and professional clinical practice to ensure consistency of examination and caries diagnosis. The mean Kappa value used to test the reliability of intra-examiner was > 0.8. Height (kg) and weight (m) were measured sequentially for each child prior to the oral examination. The body mass index (BMI) was calculated as body weight divided by the square of height.

### 2.3 Sample collection

The saliva collection of children was performed from 9: 00 to 11: 00 am. All participants were asked to refrain from eating food or drinking for at least 2 hours, without tooth brushing the night before. Before collecting saliva, the pH value of children’s oral saliva was measured by a dentist using pH strips (MColorp Hast, Merck). Each child was directed to spit saliva into an empty and sterile centrifuge tube until 2ml unstimulated saliva sample were collected. All samples were transported to the laboratory immediately and stored in a freezer at -80°C before DNA extraction.

### 2.4 16s rRNA gene sequencing methods

#### 2.4.1 DNA extraction

Total genomic DNA samples were extracted from the saliva using the OMEGA Soil DNA Kit (M5635-02) (Omega Bio-Tek, Norcross, GA, USA), following the manufacturer’s instructions, and stored at -20°C prior to further analysis. The quantity and quality of extracted DNAs were measured using a NanoDrop NC2000 spectrophotometer (Thermo Fisher Scientifific, Waltham, MA, USA) and agarose gel electrophoresis, respectively.

#### 2.4.2 16S rRNA gene amplicon sequencing

PCR amplification of the bacterial 16S rRNA genes V3–V4 region was performed using the forward primer 338F (5’-ACTCCTACGGGAGGCAGCA-3’) and the reverse primer 806R (5’-GGACTACHVGGGTWTCTAAT-3’). Sample-specific 7-bp barcodes were incorporated into the primers for multiplex sequencing. The PCR components contained 5 μl of buffer (5×), 0.25 μl of Fast pfu DNA Polymerase (5U/μl), 2 μl (2.5 mM) of dNTPs, 1 μl (10 uM) of each Forward and Reverse primer, 1 μl of DNA Template, and 14.75 μl of ddH2O. Thermal cycling consisted of initial denaturation at 98°C for 5 min, followed by 25 cycles consisting of denaturation at 98°C for 30 s, annealing at 53°C for 30 s, and extension at 72°C for 45 s, with a final extension of 5 min at 72°C. PCR amplicons were purifified with Vazyme VAHTSTM DNA Clean Beads (Vazyme, Nanjing, China) and quantifified using the Quant-iT PicoGreen dsDNA Assay Kit (Invitrogen, Carlsbad, CA, USA). After the individual quantification step, amplicons were pooled in equal amounts, and pair-end 2 x 250 bp sequencing was performed using the Illlumina NovaSeq platform with NovaSeq 6000 SP Reagent Kit (500 cycles) at Shanghai Personal Biotechnology Co., Ltd (Shanghai, China).

#### 2.4.3 Sequence analysis

Microbiome bioinformatics were performed using QIIME2. Briefly, raw sequence data were demultiplexed using the demux plugin following by primers cutting with cutadapt plugin. Sequences were then quality filtered, denoised, merged and chimera removed using the DADA2 plugin. Non-singleton amplicon sequence variants (ASVs) were aligned with mafft and used to construct a phylogeny with fasttree2. Alpha-diversity metrics (Chao1, Observed species, Shannon, Simpson, Faith’s PD, Pielou’s evenness and Good’s coverage), beta diversity metrics (Bray-Curtis and unweighted UniFrac) were estimated using the diversity plugin with samples. Taxonomy was assigned to ASVs using the classify-sklearn naiüve Bayes taxonomy classifier in feature-classifier plugin against the HOMD_16S database (Human Oral Microbiome Database).

#### 2.4.4 Bioinformatics and statistical analysis

Sequence data analyses were mainly performed using QIIME2 and R packages (v3.2.0). ASV-level alpha diversity indices, such as Chao1 richness estimator, Observed species, Shannon diversity index, Simpson index, Faith’s PD, Pielou’s evenness and Good’s coverage were calculated using the ASV table in QIIME2, and visualized as box plots. ASV-level ranked abundance curves were generated to compare the richness and evenness of ASVs among samples. Beta diversity analysis was performed to investigate the structural variation of microbial communities across samples using Bray-Curtis metrics and Unweighted UniFrac distance metrics and visualized *via* principal coordinate analysis (PCoA). The significance of differentiation of microbiota structure among groups was assessed by PERMANOVA using QIIME2. The taxonomy compositions and abundances were visualized using MEGAN. LEfSe (Linear discriminant analysis effect size) was performed to detect differentially abundant taxa across groups using the default parameters. The Spearman test method was used to analyze the correlation between biomarkers of group H and partial indicators related to caries activity in all samples, and redundancy analysis (RDA) and associated heat map were used to represent. Microbial functions were predicted by PICRUSt2 upon MetaCyc (https://metacyc.org/) databases.

Statistical analysis of the questionnaire data was performed using SPSS software (v26.0, IBM Statistics, Chicago, IL, USA). The one-way ANOVA was utilized to compare the age, BMI, the dmft scores and the pH value between the study groups. The Spearman rank correlation was used to analyze the correlation between CAT value and dmft in preschool children. Differences in the variables in the questionnaire were analyzed using the Chi-square test and the Kruskal-Wallis H test. All statistical tests were two-tailed with a statistical significance level of *P*<0.05.

## 3 Results

### 3.1 Demographics and oral health-related questionnaire survey

A total of 90 children aged 3-5 years were enrolled in this study. The numbers of high, medium and low caries activity children were 30 in each group, among which 40 were male (44.4%) and 50 were female (55.6%). There was no significant difference in gender among the three groups of CA (*P* > 0.05), and the average age was 4.08 ± 0.78 years old.

The mean dmft scores of children with high, medium and low CA were 9.07 ± 4.16, 5.73 ± 4.73 and 1.97 ± 2.48, respectively. There was a positive correlation between dmft score and CA in children (r=0.596, *P* < 0.01). The age and mean dmft score of children in group H were significantly higher than those in group M and group L (*P* < 0.05). There were significant differences in the proportion of children in the H group who consumed sugary beverages frequently compared with children in the M and L groups (*P* < 0.05). No significant differences were found among the CA groups regarding gender, Body Mass index (BMI), salivary pH, and oral health behavior (**Table 1**).

**Table 1:** Demographics of study population and analysis of related influencing factors of high CA.

Variable/Index	Group H	Group M	Group L	*P*-value
Sex				0.835
Male	12(40.0)	14(46.7)	14(46.7)	
Female	18(60.0)	16(53.3)	16(53.3)	
**Age(years)**	4.33 ± 0.66	4.10 ± 0.76	3.80 ± 0.85	0.028*
**BMI**	15.47 ± 1.93	15.41 ± 1.80	15.78 ± 2.32	0.533
**dmft**	9.07 ± 4.16	5.73 ± 4.73	1.97 ± 2.48	0.000**
**pH value**	6.76 ± 0.14	6.83 ± 0.15	6.83 ± 0.31	0.370
Mother’s delivery mode				0.614
Caesarean birth	9(30.0)	14(46.7)	13(43.3)	
natural delivery	21(70.0)	16(53.3)	17(56.7)	
Feeding methods within 6 months after birth				0.678
Breastfed	22(73.3)	24(80.0)	20(66.7)	
Mixed feeding	4(13.3)	3(10.0)	7(23.3)	
Artificially fed	4(13.3)	3(10.0)	3(10.0)	
Brushing frequency per day				0.137
≥ 2 times/day	4(13.3)	8(26.7)	1(3.3)	
1 time/day	24(80.0)	18(60.0)	22(73.3)	
< 1 time/day	2(6.7)	4(13.3)	7(23.3)	
Tooth brush duration				0.769
< 2 minutes	12(40.0)	13(43.3)	11(36.7)	
≥ 2 minutes	18(60.0)	17(56.7)	19(63.3)	
Frequency of sugary drink intake				0.001**
<1 time/day	3(10.0)	16(53.3)	6(20.0)	
1 time/day	7(23.3)	7(23.3)	5(16.7)	
≥2 times/day	19(63.3)	2(6.7)	3(10.0)	
Never	1(3.3)	5(16.7)	16(53.3)	
Frequency of sweets/snacks intake				0.695
<1 time/day	9(30.0)	6(20.0)	10(33.3)	
1 time/day	15(50.0)	12(40.0)	10(33.3)	
≥2 times/day	4(13.3)	11(36.7)	10(33.3)	
Never	2(6.7)	1(3.3)	0(0.0)	
**Fluoride toothpaste**				0.708
Yes	5(16.7)	2(6.7)	9(30.0)	
No	12(40.0)	11(36.7)	16(53.3)	
Unknown	13(43.3)	17(56.6)	5(16.7)	

*P<0.05.

**P<0.01.

### 3.2 Features of salivary microbiomes and alpha diversity analysis

**Figure 1:**
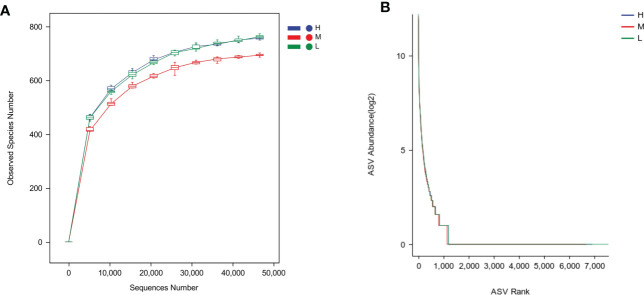
Oral bacterial community structure differs among groups. **(A)** Rarefaction curves, **(B)** Rank abundance curves. The H, M and L groups are colored in blue, red and green, respectively.

After quality filtering, Illumina Noveseq sequencing yielded a total of 7,298,614 sequences from 90 saliva samples, with an average of 81,096 reads. A total of 2,252,242 sequences were obtained from 30 saliva samples in group H, 75,075 sequences per sample for analysis, 2,297,599 sequences in group M, 76,587 sequences per sample and 2,748,773 sequences in Group L, 91,626 sequences per sample. The sequence length distribution ranged from 48 to 442bp, with an average length of 425bp per sequence. The ecological characteristics of oral saliva microbiota in the H, M and L groups were analyzed by 16S rRNA gene amplicon sequencing. The Good’s coverage index of all ASVs was approximately 0.99 ± 0.001 (**Table 2**). The rarefaction curve in **Figure 1A** showed that the samples among the three groups of different CA gradually reached a plateau with the increasing sequencing volume, which was sufficient to reflect the diversity contained in the current samples, and can comprehensively evaluate the abundance of the whole microbial community. The species richness in group M was slightly lower than that in groups H and L. The rank abundance curves reflected the steep and similar broken lines of the three groups of samples, indicating that the abundance differences among ASVs in the community were large. However, most species had low abundance and showed a “long tail distribution” (**Figure 1B**). Chao1 and Observed species indices were used for richness of the salivary microbiota, Shannon and Simpson indices for diversity, and Pielou’s evenness index to characterize evenness. As shown in **Table 2**, there was no significant difference in alpha diversity among the three groups of H, M, and L. There was also no significant difference in alpha diversity among the three groups with different caries status (Group by dmft) (**Table S1**).

**Table 2:** Comparison of species alpha diversity based on ASV levels among different CA groups.

Group	Good’s coverage	Pielou’s evenness	Richness estimator		Diversity index	
			Chao1	Observed species	Shannon	Simpson
**H**	0.99 ± 0.001	0.65 ± 0.03	756.77 ± 220.03	624.10 ± 181.29	6.01 ± 0.39	0.96 ± 0.01
**M**	0.99 ± 0.001	0.64 ± 0.04	694.52 ± 230.64	568.84 ± 179.94	5.80 ± 0.45	0.95 ± 0.21
**L**	0.99 ± 0.001	0.66 ± 0.03	761.62 ± 309.06	616.94 ± 267.05	6.02 ± 0.43	0.96 ± 0.13
***P*-value**	0.838	0.139	0.483	0.417	0.203	0.280

### 3.3 Salivary microbiota composition and beta diversity analysis

**Figure 2:**
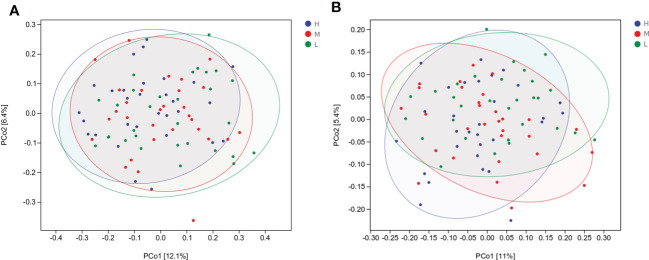
Microbial community composition in H, M and L groups evaluated by principal coordinate analysis (PCoA) based on beta diversity, including Bray_Curtis distances **(A)**, and Unweighted UniFrac distances **(B)**.

To compare microbial community composition of saliva samples from children among different CA groups (Group H, M, and L) and different dmft groups (Group CF, LC, and HC), beta diversity was calculated based on Bray-Curtis and Unweighted UniFrac distances. The oral microbes from the three groups were clustered according to community composition and then visualized by principal coordinate analysis (PCoA). From the sample score table of the main coordinate (Appendix 1), the principal coordinates of the two points H9 and H18 with the biggest difference in group H differ by 0.587212, while the scores of the two points L21 and L26 with the biggest difference in group L differ by 0.605233, which was larger than that of group H. Meanwhile, compared with group L, the projection distance of samples in group H on the coordinate axis of PCoA 1 was closer, which indicated that the community composition in group H was relatively more clustering and conservative, while the community composition in group L was relatively scattered and complex (**Figures 2A, B**).

PERMANOVA’s results confirmed significant differences of microbial community composition among the three groups with different CA. Among children with different CA, there was a significant difference in the composition of salivary microbial communities between group H and group L (*P* < 0.05). The composition of oral microbial communities in children with different caries status was statistically different (*P* < 0.05) (**Table S2**). There were significant differences in the composition of microbial communities between CF and LC groups (*P* < 0.05) and between CF and HC groups (*P* < 0.05). However, there was no significant difference in microbial community composition between LC group and HC group (*P* > 0.05).

### 3.4 Analysis of difference in oral bacterial taxa

**Figure 3:**
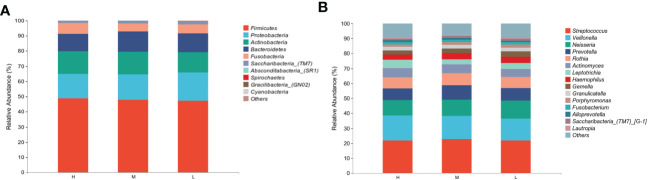
Comparison of relative abundance of different taxa among different CA groups. **(A)** Top 10 in abundance at the phylum level, **(B)** Top 15 in abundance at the genus level.

A total of 15 phyla, 34 classes, 61 orders, 113 families, and 215 genera were detected in all samples from three groups of different CA. At the phylum level, the composition and relative abundance of saliva samples were similar, although slight differences existed among the groups H, M, and L (**Figure 3A**). 99.82% of the sample microflora was composed of the following six phyla with relatively high abundance, which were ranked in descending order: *Firmicutes* (48.00%), *Proteobacteria* (17.08%), *Actinobacteria* (14.43%), *Bacteroidetes* (12.54%), *Fusobacteria* (5.99%) and *Saccharibacteria_(TMT)* (1.78%). Of the 15 phyla detected in this study, only *Absconditabacteria_(SR1)* showed significant differences among groups H, M, and L (*P* < 0.05) (**Table S3**).

In different CA groups, the most prevalent genera were *Streptococcus* (22.19%), *Veillonella* (15.51%), *Neisseria* (11.09%), *Prevotella* (8.60%), *Rothia* (7.67%), *Actinomyces* (5.78%), *Leptotrichia* (4.43%), *Haemophilus* (3.80%), *Gemella* (3.16%) and Granulicatella (2.41%), together comprising 84.64% of the total sequence (**Figure 3B**), which was consistent with the distribution of the top 15 abundant genera in the CF, LC, and HC groups (**Figure S1A**). Thirty subjects in every three CA groups (high, medium, and low) showed similar trends in salivary microbiota at the phylum and genus levels (**Figures S3A, B**). In this study, a total of 14 genera showed significant differences among the different CA groups (*P* < 0.05) (**Table S3**), but the abundance of most genera was low.

**Figure 4:**
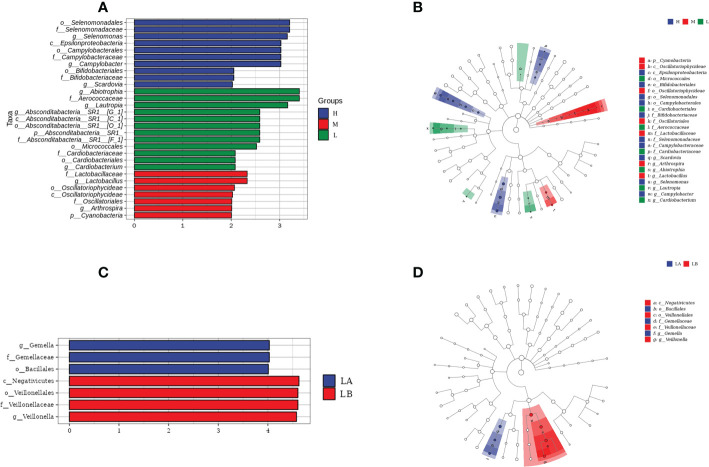
Differences in oral salivary microbiota composition among children with different CA. **(A)** Linear discriminant analysis (LDA) scores for bacterial taxa differing in abundance among different CA groups. **(B)** Cladograms generated by LEfse showing differences at phylum, class, family and genus levels among H, M and L groups. Each successive circle represents a phylogenetic level. Regions in blue indicate taxa enriched in group H, regions in red indicate taxa enriched in group M, while regions in green indicate taxa enriched in group L. **(C)** LDA scores for bacterial taxa differing in abundance between the LA and LB groups. **(D)** Cladograms generated by LEfSe indicating taxonomic differences between LA and LB groups.

LEfSe analysis was used to further analyze the bacterial community structure and highlighted the potential biomarkers of different groups, and linear discriminant analysis (LDA) was performed to estimate the difference in effect size of each taxon in the three groups (**Figures 4A, B**, **S2A, B**). Among the different CA groups, *Epsilonproteobacteria*, *Bifidobacteriales*, *Selenomonadales*, *Campylobacterales*, *Bifidobacteriaceae*, *Selenomonadaceae*, *Campylobacteraceae*, *Scardovia*, *Selenomonas*, *Campylobacter* resulted to be potential caries biomarkers in group H, while *Micrococcales*, *Cardiobacteriales*, *Aerococcaceae*, *Cardobacteriaceae*, *Abiotrophia*, *Lautropia*, *Cardiobacterium* was elected to be associated with the low CA status. *Cyanobacteria*, *Oscillatoriophycideae*, *Epsilonproteobacteria*, *Oscillatoriophycideae*, *Oscillatoriales*, *Lactobacillaceae*, *Arthrospira*, *Lactobacillus* were significantly enriched in group M (*P* < 0.05).

Among the three groups with different caries status, at the genus level, *Mobiluncis*, *Capnocytophaga*, *Abiotrophia*, *Cardiobacterium* were the potential biomarkers in the CF group and Veillonella was elevated in the LC group. *Bifidobacterium*, *Parascardovia*, *Scardovia*, *Atopobium*, *Alloprevotella*, *Butyrivibrio*, *Selenomonas*, *Leptotrichia*, *Desulfovibrio* were significantly enriched in the HC group in comparison with the CF and LC groups.

The results of the present study showed that *Scardovia* and *Selenomonas* were the potential biomarkers in both groups H and HC, while the *Abiotrophia* and *Cardiobacterium* were significantly increased in both groups L and LC.

In the L group of this study, 14 children (L1, L2, L3, L4, L5, L6, L8, L22, L24, L25, L26, L27, L28, L30) had dmft = 0, and the remaining 16 children had dental caries (1 ≤ dmft ≤ 14), with an average dmft of 4.31 ± 3.44. In group L, 14 caries-free children were defined as LA group, and 16 caries-affected children were defined as LB group. Meanwhile, the differences in microbial community structure between the LA group and the LB group were explored using LEfSe analysis. The results showed that at the genus level, the potential biomarker of the LA group was the *Gemella*, which was also significantly reduced in the LB group, and *Veillonella* was significantly enriched in the LB group (**Figures 4C, D**). In this study, 5 children in group M (M6, M7, M8, M12, M17) were found to have dmft = 0, which were defined as group MA; The remaining 25 children, whose mean dmft score was 6.88 ± 4.33, were defined as the MB group. LEfSe analysis showed that *Leptotrichia* and *Corynebacterium* were significantly enriched in the MB group, but no bacteria were significantly enriched in the MA group (**Figure S1B**). There were no caries-free children in group H of this study.

### 3.5 Correlation analysis between some indexes and different biomarkers and establishment of diagnostic model

**Figure 5:**
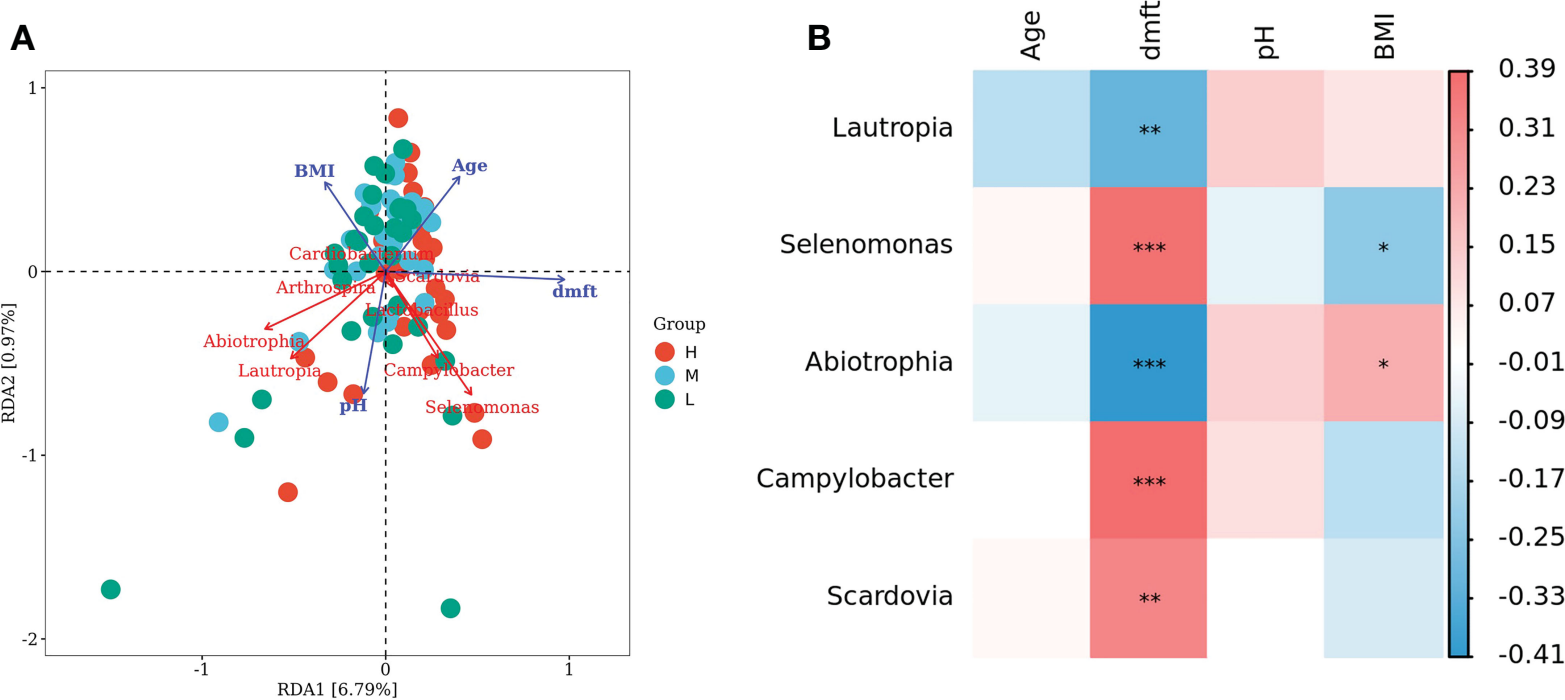
Connections between oral salivary microbiota and some indexes. **(A)** Redundancy analysis (RDA) of age, dmft, BMI, pH value and biomarkers between different CA groups. Each point represents a sample, different colored points belong to different groups. Blue arrows represent different indexes, red arrows represent bacterial genera, the angle between arrows represents the correlation between them. **(B)** Heatmap of association between age, dmft, BMI, pH value and biomarkers among different CA groups. Correction trait values are shown on the right, with default red representing a positive correlation, blue representing a negative correlation, and color depth indicating the strength of the correlation, *P < 0.05, **P < 0.01, ***P < 0.001.

In the redundancy analysis (RDA), the length of dmft rays was the longest, indicating that it had the most significant influence on the structure of oral microflora. *Selenomonas* and *Campylobacter* had a significant positive correlation with dmft (*P* < 0.001), and their relative abundance was higher in group H; There was a significant negative correlation between the *Abiotrophia* and *Cardiobacterium* and dmft (*P* < 0.001) (**Figures 5A, B**). These results suggest a correlation between the structural alterations of salivary microbiota with different caries activity and the oral state of the host.

**Figure 6:**
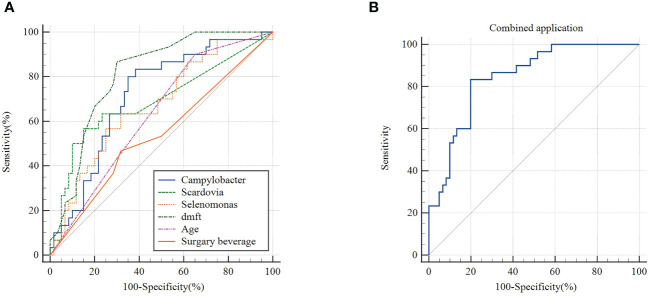
**(A)** ROC curve of single index (Campylobacter, Scardovia, Selenomonas, dmft, age, frequency of sugary beverage intake) in group H **(B)** ROC curve of combined application of each single index in group H.

The relevant influencing factors with significant differences in univariate analysis and potential salivary biomarkers in group H were used to evaluate the predictive value of children with high CA, as follows: (1) The Receiver operating characteristic curve (ROC) diagnostic analysis model of children with high caries activity was established by the single application of each indicator. **Figure 6A** shows that these single indicators have certain evaluation value. The Area under Curve (AUC) of age, dmft score, frequency of consuming sugary beverage, Scardovia, *Selenomonas* and *Campylobacter* were 0.631, 0.810, 0.541, 0.690, 0.671 and 0.717, respectively. (2) Comprehensive regression was performed on each of the individually applied indicators, and the evaluation value of the combined application of each indicator in the prediction of children with high CA showed that the AUC of the comprehensive indicator was 0.842, which was higher than each of the single indicators in evaluation efficiency (**Figure 6B**).

### 3.6 Functional profiles of salivary microbiota in different CA groups

**Figure 7:**
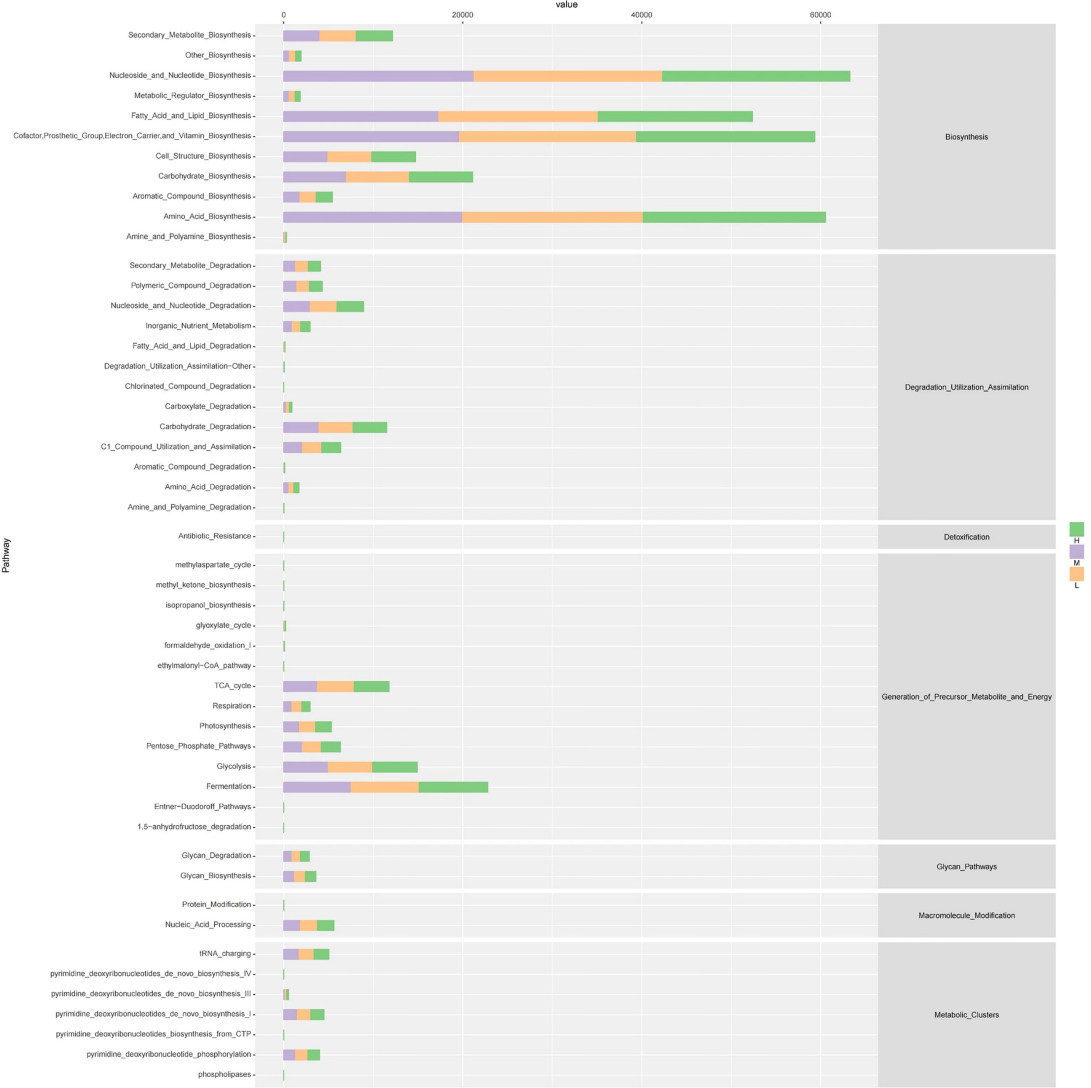
The outline functional analysis among H, M and L groups.

**Figure 8:**
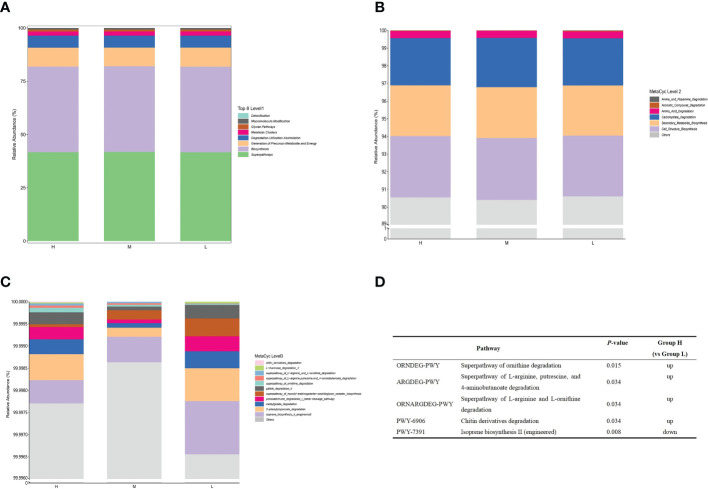
Functional profiles of salivary microbiome among different CA groups. The comparison of functional pathways at the MetaCyc level 1 **(A)**, level 2 **(B)**, and level 3 **(C)**. **(D)** The significantly altered functional pathways between the H and L groups.

Since the microbial composition of saliva samples differed between different CA groups, PICRUSt2 was used to compare the existing 16SrRNA gene sequencing data to predict the functional potential of oral microorganisms. **Figure 7** shows the distribution of microbiota abundance in the six categories of metabolic pathways among the different CA groups. We found that the great majority of salivary microbial functions belonged to categories including Biosynthesis, Degradation/Utilization/Assimilation and Generation of Precursor Metabolite and Energy, whilst functional differences could not be provided in this figure pattern. To obtain a systematic understanding, the metabolic pathways at the 1/2/3 level were analyzed and predicted by Metacyc database. At level 1 and level 2, there were no significant differences between different CA groups (**Figures 8A, B**). When down to level 3, there were 11 metabolic pathways differentially regulated among the three groups (**Figure 8C**). Compared with the group L, the superpathway of ornithine degradation (ORNDEG-PWY), superpathway of L-arginine, putrescine, and 4-aminobutanoate degradation (ARGDEG-PWY), superpathway of L-arginine and L-ornithine degradation (ORNARGDEG-PWY), chitin derivatives degradation (PWY-6906) were up-regulated in the group H children, while isoprene biosynthesis II (engineering) (PWY-7391) was down-regulated in the group H children (*P* < 0.05) (**Figure 8D**).

## 4 Discussion

To date, the present study is the first to explore the characteristics of oral salivary microbial composition and its related influencing factors in preschool children with different CA states. It was found that children with different CA and dmft scores had differences in the composition of salivary microbiota in this study. A total of 14 genera were significantly different among the three groups of children with different CA. Secondly, in the low caries activity group, different potential biomarkers in the oral saliva of caries-free (dmft = 0) and caries-affected children (dmft > 0) were analyzed in this study, which to some extent explained the phenomenon that CAT values of ECC children with higher dmft scores may still be in the relatively safe range. Thirdly, we also explored the related factors affecting CA in preschool children, and analyzed the correlation between CA-related indicators including age, dmft, BMI, and saliva pH and significantly enriched microbial communities in different CA groups. In addition, based on the results of this study, a comprehensive diagnostic model that can be used to screen children at high risk of caries was established, which provided the basis and ideas for accurately predicting and blocking the occurrence and development of ECC. Fourthly, we also found that there were 11 metabolic pathways with significant differences between different CA groups. Compared with group L, the L-arginine degradation pathway in group H was significantly active, suggesting that this metabolic pathway may be related to the occurrence and development of childhood caries and provides a basis for the targeted blocking of ECC to a certain extent. Similar to previous studies, the results of this study also show that the CAT values of children from 3 to 5 years old have a positive correlation with dmft scores in this study, and the application of Cariostat caries activity test is one of the reliable means for the prediction of children’s ECC.

With the rapid development of bioinformatics methods, about 280 oral microorganisms can be identified by traditional culture methods (Dewhirst et al., 2010). High-throughput 16S rRNA sequencing, as a more sensitive and culture-independent method, can better understand the bacteria and oral microorganism-related diseases and provide new insights into the composition and structure of microbial communities (Yang et al., 2012). With the rapid development of bioinformatics methods, compared with the traditional culture methods, which can identify about 280 oral microorganisms (Dewhirst et al., 2010), high-throughput 16S rRNA sequencing, as a more sensitive and culture-independent method, can better understand the microbial-related diseases in the oral cavity and provide new insights into the composition and structure of microbial communities (Yang et al., 2012). Although the sequences vary widely among different bacteria, the high-throughput sequencing technique can provide accurate and reliable analysis of the different hypervariable regions (V1–V9) of the bacterial 16S rRNA gene (Claesson et al., 2010), while multiple studies (Xu L. et al., 2018; Zheng et al., 2018; Claesson et al., 2010) have confirmed that, regardless of sequencing technology and quality, the V3–V4 region produces the most accurate phylogenetic allocation, providing rich information for the classification of microbial communities in human microbial samples. Therefore, the V3-V4 regions were selected for analysis of the oral salivary microbiota of children with different CA in this study to ensure the highest accuracy of analysis and adequate availability of information.

Alpha diversity refers to the indicators of species richness, diversity and uniformity in locally homogeneous habitat, also known as within-habitat diversity. Xiao et al. (Xiao et al., 2016) believed that the microbial diversity in dental plaque in a healthy state exceeded that in dental caries state and decreased with the severity of dental caries. The alpha diversity results of this study indicated that there were no significant differences in species richness, diversity, and evenness among children with different CA and caries status. This may be due to the good homogeneity of the population included in this study, that is, all children from the same area had similar living environment, cultural customs, diet and other lifestyles. This result is also similar to a 2-year cohort study that observed the dynamic changes in the salivary microbiota of primary dental caries in caries-free and caries-affected preschool children (Xu L. et al., 2018).

Bray-Curtis distance analysis considers not only the presence or absence of species, but also the ebb and flow of microbial abundance. However, Unweighted UniFrac distance analysis is a qualitative measure of beta diversity of microbial communities and can detect the presence of phylogenetic relationships between dissimilar community members. Therefore, in order to obtain more reliable results, the above two distance analyses were adopted to evaluate the differences between microbial communities in this study. The results of beta diversity analysis revealed significant differences in salivary microbial communities among different CA groups, indicating that compared with groups M and L, the salivary microbial community of children in group H was more similar to others within the H group. Concurrently, we also found that there were significant differences in the composition of salivary microbiota among children with different caries status. The above results suggested that the composition of salivary microbial community was highly correlated with CA, and monitoring the composition and change of microbial community might be an important means to block the development of ECC.

This study found that the most dominant phylum in salivary microbiota of different CA groups was *Firmicutes*, *Proteobacteria*, *Actinobacteria*, *Bacteroidetes* and *Fusobacteria*, which were similar to the results of previous studies on the oral flora of children with ECC (Zhang et al., 2015; Xiao et al., 2016). Among them, *Firmicutes* had the highest relative abundance (47.87%) in group H, which was also proved to be the prevalent phylum in supragingival plaque of children with severe early childhood caries (SECC) (Agnello et al., 2017). The study found that the abundance of *Actinobacteria* and *Fusobacteria* was higher in caries-free children (Agnello et al., 2017), while the results of this study showed that the abundance of these two phyla was similar in different CA groups, which was different from the results of the above study. This finding suggested that the oral microbiota affecting CA in children might be different from that affecting ECC in children.

Among the top 15 genera in the most abundant bacterial taxa, only *Lautropia* showed significant difference between different CA groups, and its abundance in group L was relatively high. *Lautropia*, *Abiotrophia* and *Cardiobacterium* were significantly enriched in group L, but decreased in group H. Furthermore, we observed that an increased level of *Abiotrophia* and *Cardiobacterium* in the CF group associated with oral health. There was a negative relationship between *Abiotrophia* and dmft score in the present study. In previous studies, *Lautropia* was reported to be closely related to health status (Chen et al., 2021), *Cardiobacterium* was detected in the dark spots of caries-free patients (Çelik et al., 2021), and *Abiotrophia* was also found to be beneficial to dental health (Muhoozi et al., 2022). All the above three taxa had some relationship with oral health of non-ECC children, which was similar to the results of our study. This suggested that the three bacterial genera might be associated with low CA status in children, but their abundance values were low (< 1% abundance) in this study. Thus, more studies are needed to investigate the cariogenic roles of these three bacteria in CA and ECC.

An increasing amount of evidence supports the idea that dysbiosis of oral microbial community is an important cause of ECC (Xu H. et al., 2018; Zheng et al., 2018; Banas and Drake, 2018). Some bacteria with highly acidogenic and acid-tolerant characteristics are considered to be the “core microbiota” associated with ECC, including *Streptococcus mutans*, *Lactobacillus*, *Neisseria* and *Veillonella*. The results of this study showed that the relative abundance of the genus *Streptococcus* in different CA groups was the highest (the average abundance of the three groups was 22.19%) but no obvious difference was found, and there was no significant dissimilarity among CF, LC and HC groups. This finding suggested that *Streptococcus* was not the dominant genera in group H and group HC, which was similar with the previous study that *S. mutans* was not the dominant bacterium in the oral microbial community of children with caries and existed in large amounts in the oral cavity of children without caries. The possible explanation is that *Streptococcus* contains many species, such as *Streptococcus mutans*, *Streptococcus sanguis*, *Streptococcus oralis*, etc. These *streptococcus* species show different abundance due to various roles in the development of dental caries. Based on the results of this study, we speculated that although the relative abundance of *Streptococcus* spp. in different CA groups was high, it might not be the key flora causing CA fluctuation, indicating that *Streptococcus* spp. might be cariogenic under certain conditions or involved in the occurrence of ECC together with other more sensitive bacteria.

The proportion of *Lactobacillus* in the oral cavity of children aged 3 months can be as high as 46% in a previous study (Lif Holgerson et al., 2015). Zheng et al. (Zheng et al., 2018) believed that *Lactobacillus* in sugingival dental plaque could hydrolyze proteins into amino acids and dipeptides, which was conducive to the growth of *Streptococcus* mutans in the oral cavity and was significantly related to the occurrence and development of ECC. Xu et al., 2021a held the opposite opinion, believing that *Lactobacillus* in saliva is beneficial bacteria and plays a critical role in regulating local microbial ecological balance. The results of this study showed that the genus *Lactobacillus* was enriched in the M group, suggesting that *Lactobacillus* may be the potential cariogenic bacteria in the M group and may be the dominant microorganism leading to the development of children’s oral caries activity from low level to high level. Blocking the proliferation and colonization of *Lactobacillus* may also reverse CA from high to low in children. In addition, it is worth noting that among the 90 samples in this study, this genus was not detected in the saliva of 19 samples (21.1%), of which 7 subjects had dmft=0, and the remaining 12 children had an average dmft score of 5.58 ± 3.23. Our results again suggest that *Lactobacillus* was not present in the oral salivary microbiota of all children, and the oral status of the 19 children in whom no *Lactobacillus* was found in saliva was not all caries-free. Jiang et al. (Jiang et al., 2016) also found that although the abundance of *Lactobacillus* in saliva in the caries group was significantly higher than that in the non-caries group (0.06% vs. 0.002%, respectively), there was almost no *Lactobacillus* in all samples, which was in line with the results of this study. Although the high-throughput sequencing technology was used for the first time to explore the distribution characteristics and correlation of *Lactobacillus* in saliva of children with different CA, the sequencing technology applied in this study was limited to the detection of bacteria at the genus level; Moreover, there were differences in the detection rates of *Lactobacillus* in different sites, such as saliva (88.51%), supragingival plaque (87.36%), subgingival plaque (89.66%), soft tissue (88.51%), and tongue (89.66%) (Gizani et al., 2009). This also needs to be explored with more research due to the possible impact of divergences in colonization sites on CA and ECC in children. In addition to the genus *Lactobacillus*, *Arthrospira* was significantly enriched in the M group compared with the H and L groups. Interestingly, *Arthrospira* was not previously detected in children with ECC, suggesting that attention should be paid to its possible role in the fluctuation of caries activity.

The abundance of the genus *Leptotrichia* and *Corynebacterium* was found to be relatively increased in the MB group in the present study. Chen et al. (Chen et al., 2021) considered that *Leptotrichia* had a high ability to metabolize carbohydrates, which was closely related to the high prevalence of dental caries, and it might be a potential cariogenic biomarker. On the contrary, *Leptotrichia* was considered to be related to oral health of children and *Corynebacterium* was detected in children with caries in the study by Zhang et al., 2015. Since the relationship between *Leptotrichia* and *Corynebacterium* and CA had not been reported in previous studies, the results of this study suggested that these two bacteria may play an important role in the fluctuation of caries activity from low to high in children. With the changes of host oral environment and other factors, the relative abundance of *Leptotrichia* and *Corynebacterium* in saliva also changes. Therefore, whether blocking the colonization of these two bacteria or inhibiting their acid production can reverse the transformation of CA status to low level needs to be further studied and tested.

Previous *in vitro* studies showed that co-culture of *S. mutans* and *V. parvula* resulted in a 50% to 150% increase in sucrose-dependent biofilm mass compared to *S. mutans* alone, while *V. parvula* alone did not form an *in vitro* biofilm (Abram et al., 2022), indicating that *S. mutans* and *V. parvula* have a synergistic effect, and *Streptococcus* can provide adhesion sites and nutrient sources for *Veillonella*. The results of the meta-analysis by Dame-Teixeira et al., 2021 revealed that *Veillonella* spp. was more abundant in individuals suffering with ECC in comparison with caries-free controls. This study found that the abundance of *Veillonella* spp. was relatively augmented in both the LC group and the LB group. As one of the cariogenic bacteria in the oral cavity, it was enriched in children with low CA, suggesting that the cariogenic effect of Veillonella was limited, and it might be the key flora to maintain the low CA state in saliva and inhibit the occurrence and development of ECC. Previous studies described that using lactic acid rather than carbohydrates as the energy source, *Veillonella* can convert the metabolic strong and non-volatile lactic acid into weak and volatile acetic acid and propionic acid (Liu et al., 2020). In addition, *Veillonella* also has the ability to produce nitrite (NO2^-^) by reducing nitrate (NO3^-^) (Wicaksono et al., 2020). Nitrite (NO2^-^) has been reported to inhibit the production of dental plaque acid (Yamamoto et al., 2017). Hence, how *Veillonella* plays a role in the occurrence and development of CA and ECC in children needs needs more evidences and further research. Furthermore, we also found that *Gemella* were significantly enriched in the LA group, while Xu et al., 2021b also considered it to be one of the core microflora in the oral saliva of caries-free preschool children, suggesting that *Gemella* may be a beneficial microflora to maintain oral health of children.

Our study identified potential biomarkers associated with dmft scores between different CA groups. The biomarkers of group H, *Selenomonas* and *Campylobacter*, were positively correlated with dmft scores, that is, in general, if a child has a higher dmft score, the acidic environment of the host’s mouth is more conducive to the survival of the biomarkers of group H. Conversely, biomarkers in group L negatively correlated with dmft, *Abitrophia* and *Lautrophia*, may be relatively abundant in the oral cavity of healthy children. To some extent, these results not only reflect that Cariostat caries activity test can effectively predict the caries status of preschool children, but also indicate that the oral microbiota of different children may also show dynamic changes with the changes of CA and dmft scores. To explore the mechanism of the relationship among CA, dmft and oral microbiota is also the key to revealing the occurrence of ECC.

*Scardovia* is one of the seven genera of the *Bifidobacterium* family, and is a new bacterial genus isolated from the *Bifidobacterium* genus in 2002 due to the discrepancies in their genomic sequences (Jian and Dong, 2002). No previous studies have reported the association between *Scardovia* and CA. The results of this study showed that the genus *Scardovia* was significantly enriched in the H and HC groups compared with other groups, suggesting that the enrichment of *Scardovia* might be closely related to the development of high CA and ECC in children, which was in good agreement with previously described results (Kameda et al., 2020; Prabhu Matondkar et al., 2020; McDaniel et al., 2021). The reason may be that as an anaerobes, *Scardovia* can survive in mature biofilms with low oxygen concentration and metaboltize glucose to produce acid, which eventually leads to a decrease in oral pH and an increase in CAT value.

In this study, *Selenomona*s and *Campylobacter* were found to be positively correlated with dmft scores, and *Selenomonas* was enriched in H and HC groups, which might be due to the fact that it could ferment glucose and utilize lactic acid to play a role in the high caries activity and caries progression in children. In previous studies, uncultured *Selenomonas* species were associated with root caries in the elderly (Preza et al., 2008) and coronal caries in young children (Corby et al., 2005). It has been suggested that *Selenomonas* may be an early and persistent biomarker of the development of the oral environment to a cariogenic state because of its enrichment before caries (Grier et al., 2021). However, a study in children under 30 months showed the opposite result, that *Selenomonas*, *Capnocytophaga*, and *Leptotricia* were more related to the caries-free samples (Xu et al., 2014). Given the results of previous studies, we hypothesized that the distribution and pathogenicity of *Selenomonas* in the oral cavity may be closely related to the age of the host. In this study, the subjects were all over 30 months old, and the results showed that *Selenomonas* was enriched in the H and HC groups, suggesting that *Selenomonas* in the oral cavity may show its potential cariogenicity with age and changes in oral microbiota colonization. When it comes to *Campylobacter*, it was considered to be a common pathogenic bacterium. *Campylobacter* is associated with intestinal diseases, and its detection rate in the intestinal tract of patients with Inflammatory Bowel Disease (IBD) was higher than that of healthy people (Qi et al., 2021). *Campylobacter* and *Selenomonas* used organic acids as energy sources and were closely associated with dental caries (Aas et al., 2008; Nagai et al., 2020). In contrast, *Campylobacter* reportedly was more abundant in the oral cavity of healthy individuals than caries patients, and that increased abundance reduced the risk of caries (Lif Holgerson et al., 2015; Xu et al., 2021a). *Scardovia*, *Selenomonas* and *Campylobacter* are all Gram-negative bacteria, and the enhancement of their acidogenic ability often leads to the increase of CA, so they may have a close relationship with ECC as biomarkers in group H and group HC.

A study by Pierce et al. (Pierce et al., 2019) showed that age is an important risk factor for ECC and has a significant correlation with the development of SECC. Children’s CAT values increased with age, as reported by other study groups (Nishimura et al., 2008; Xuan et al., 2017). In this study, the average age of children in group H was significantly higher than that in the M and L groups, and the differences among the three groups were significant, which might be due to the fact that the older children had more opportunities to be exposed to cariogenic microorganisms and cariogenic oral environment, and thus increased the risk of high caries activity. The study results of Imes et al. (Imes et al., 2021) showed that unhealthy dietary habits such as frequent or excessive intake of sugar-sweetened beverages were significantly related to the occurrence of ECC. The reason may be that frequent sugar intake causes microorganisms in the oral cavity to produce organic acids and exopolysaccharides by metabolize carbohydrates. When the pH value of the microenvironment in the oral cavity decreases, the CAT value tends to increase.

In this study, ROC curve analysis showed that dmft, age, sugary beverages, *Scardovia*, *Selenomonas*, and *Campylobacter* had certain predictive value, and the combination of multiple factors had higher diagnostic efficiency, which had reliable application value for the identification of high CA. In terms of the overall trend of functional prediction, we found that there were significant differences in metabolic pathways in the saliva microflora among the three groups. Compared with group L, the L- arginine degradation pathway involved in group H was significantly up-regulated. He et al. (He et al., 2016) found that L- arginine at 1.5% could regulate the development of cariogenic biofilm by arginolytic bacteria. Arginolytic bacteria such as S.gordonii can process arginine to yield ammonia through the arginine deiminase pathway (ADS), thereby promoting the neutralization of glycolytic acid and increasing the pH in oral biofilm. Liu et al. (Liu et al., 2022) demonstrated that arginine could reduce the growth of *S. mutans* biofilm and acid production by inhibiting glycolysis, the metabolism of amino sugar and nucleotide sugar, and the synthesis of peptidoglycan. Arginine, as an important substrate for alkali-producing metabolism of oral bacteria, is an important mechanism for determining the acid-base balance of dental plaque and regulating the microecological balance of dental plaque biofilm (Bijle et al., 2020). The upregulation of the L- arginine degradation pathway suggested that the group H had potential cariogenicity.

This study aims to establish a comprehensive diagnostic model to screen children at high risk of CA and provide new ideas for preventing the occurrence of ECC. Our results suggest whether it is possible to use specific biomarkers in the oral cavity of children with high CA as targets, take appropriate measures to change the oral microenvironment, and combine with personalized nutrition and hygiene management to achieve the purpose of blocking ECC. However, this study is a cross-sectional study, which may only show the microbial situation at a certain point in time, and does not necessarily accurately and comprehensively reflect the complete oral microenvironment and determine when the oral flora changes from low CA state to high CA state. Secondly, the number of samples included is small, so more and larger-scale cohort studies combined with multi-omics analysis are needed in the future to further observe the dynamic relationship between oral microbiota, CA and the occurrence of ECC.

## 5 Conclusion

There are significant differences in salivary microbial communities among children with different CA and caries status, and the specific biomarkers are various among children with high, medium and low CA. *Scardovia*, *Selenomonas*, and *Campylobacter* may be potential cariogenic bacteria in the saliva of children with high CA, and *Abiotrophia*, *Cardiobacterium* and *Lautropia* are associated with children with low CA. Among them, *Scardovia* and *Selenomonas* may also play an important cariogenic role in the mouth of children with high dmft scores, while *Abiotrophia* and *Cardiobacterium* may be a potentially beneficial bacteria to maintain low dmft scores in children’s oral cavity. The microorganisms significantly enriched in different CA states are closely related to the microflora in the oral saliva of children with different dmft. The combination of dmft score, age, frequency of sugary beverage intake, S*cardovia*, *Selenomonas*, and *Campylobacter* has good predictive value for high CA in children. This study provides a comprehensive diagnostic model to screen children with high caries risk and a new idea for blocking the etiology of ECC.

## Data availability statement

The datasets presented in this study can be found in online repositories. The names of the repository/repositories and accession number(s) can be found below: NCBI, SRP420678, PRJNA930569.

## Ethics statement

The studies involving human participants were reviewed and approved by Ethics Committee of Dental Hospital of Hebei Medical University (Shijiazhuang, HeBei, China). Written informed consent to participate in this study was provided by the participants’ legal guardian/next of kin.

## Author contributions

HS conceived and designed the study. ZM, XL, YW, and MX trained the data collectors. XL, YW, ZM, JC, YT and ZL analyzed and reconciled the data. XL and MX contributed to the preparation of manuscript, which was checked and revised by HS. All authors contributed to the article and approved the submitted version.
