# Supplementary material for: Correlation between caries activity and salivary microbiota in preschool children
Source: Front Cell Infect Microbiol. 2023 Apr 11;13:1141474. doi: 10.3389/fcimb.2023.1141474 (PMC10126836; doi:10.3389/fcimb.2023.1141474)
Supplement: Supplementary file 2 [file DataSheet_2.doc]

***Supplementary Material***

**Correlation between caries activity and salivary microbiota in preschool children**

**Xiuyan Lin, Yuan Wang, Zhe Ma, Meng Xie, Zhuo Liu, Jinghui Cheng, Yuzhao Tian and Hong Shi***

***Correspondence:**

Hong Shi

[shihong@hebmu.edu.cn](mailto:shihong_hb@163.com)

**1.Supplementary Tables**

Table S1. Comparison of species alpha diversity based on ASV levels among different caries status groups

| **Group** | **Good’s coverage** | **Pielou’s evenness** | **Richness estimator** | | **Diversity index** | |
| --- | --- | --- | --- | --- | --- | --- |
|  |  |  | **Chao1** | **Observed species** | **Shannon** | **Simpson** |
| **CF** | 0.99±0.001 | 0.65±0.04 | 643.44±201.34 | 520.31±146.99 | 5.82±0.49 | 0.95±0.02 |
| **LC** | 0.99±0.001 | 0.65±0.04 | 732.01±172.18 | 595.89±136.22 | 5.92±0.37 | 0.95±0.02 |
| **HC** | 0.99±0.001 | 0.65±0.03 | 781.76±306.71 | 643.67±261.44 | 6.00±0.44 | 0.96±0.01 |
| ***P*-value** | 0.168 | 0.799 | 0.139 | 0.089 | 0.454 | 0.779 |

**Table S2. Analysis of microbiota difference between different CA groups and caries status groups.**

| **Distance Algorithm** | **CAT/dmft** | | **Pseudo-F** | ***P*-value** |
| --- | --- | --- | --- | --- |
|  | **Group1** | **Group2** |  |  |
| **Bray_curtis** | all | - | 1.391876 | 0.024* |
|  | H | M | 1.378229 | 0.062 |
|  | H | L | 1.660661 | 0.022* |
|  | M | L | 1.146167 | 0.196 |
|  | all | - | 1.762021 | 0.001** |
|  | CF | LC | 1.903338 | 0.006** |
|  | CF | HC | 2.612538 | 0.001** |
|  | LC | HC | 0.934636 | 0.561 |
| **Unweighted_Unifrac** | all | - | 1.527661 | 0.006** |
|  | H | M | 1.184879 | 0.139 |
|  | H | L | 2.118532 | 0.001** |
|  | M | L | 1.283489 | 0.072 |
|  | all | - | 1.521874 | 0.004** |
|  | CF | LC | 1.500299 | 0.021* |
|  | CF | HC | 2.017169 | 0.002** |
|  | LC | HC | 1.111927 | 0.206 |

* *P*＜0.05

** *P*＜0.01

Table S3. Species with significant differences at the phylum and genus levels.

| **Phylum/Genus**  **level** | **Group H** | | **Group M** | | **Group L** | | ***P*-value** |
| --- | --- | --- | --- | --- | --- | --- | --- |
|  | **Mean value** | **SD** | **Mean value** | **SD** | **Mean value** | **SD** |  |
| p_Absconditabacteria_[SR1] | 0.000610 | 0.001036 | 0.000814 | 0.001083 | 0.000410 | 0.000602 | 0.033308 |
| g_Lautropia | 0.006513 | 0.008295 | 0.005831 | 0.005235 | 0.009069 | 0.006813 | 0.011956 |
| g_Lactobacillus | 0.000566 | 0.000587 | 0.000705 | 0.000848 | 0.000322 | 0.000315 | 0.020286 |
| g_Campylobacter | 0.005535 | 0.002861 | 0.003685 | 0.002759 | 0.003828 | 0.002520 | 0.003505 |
| g_Arthrospira | 0 | 0 | 0.000202 | 0.000806 | 0.000705 | 0.000285 | 0.044347 |
| g_Abiotrophia | 0.002028 | 0.002192 | 0.004902 | 0.007424 | 0.004536 | 0.007755 | 0.001862 |
| g_Lautropia | 0.006513 | 0.008295 | 0.005831 | 0.005235 | 0.009069 | 0.006813 | 0.011956 |
| g_Cardiobacterium | 0.000181 | 0.000265 | 0.000202 | 0.000156 | 0.000417 | 0.000432 | 0.002567 |
| g_Scardovia | 0.000263 | 0.000681 | 0.000084 | 0.000188 | 0.000037 | 0.000115 | 0.002617 |
| g_Selenomonas | 0.007831 | 0.005919 | 0.004787 | 0.002866 | 0.005774 | 0.007662 | 0.022476 |
| g_Parascardovia | 0.000024 | 0.000057 | 0.000002 | 0.000012 | 0 | 0 | 0.008751 |
| g_Shuttleworthia | 0.000019 | 0.000031 | 0.000020 | 0.000091 | 0.000007 | 0.000020 | 0.001451 |
| g_Tannerella | 0.000242 | 0.000364 | 0.000132 | 0.000208 | 0.000138 | 0.000156 | 0.048515 |
| g_Bacteroidetes_[G-7] | 0.000043 | 0.000084 | 0.000054 | 0.000070 | 0 | 0 | 0.000241 |
| g_Absconditabacteria_(SR1)_[G-1] | 0.000610 | 0.001036 | 0.000458 | 0.000630 | 0.001254 | 0.001687 | 0.033309 |

1. **Supplementary Figures**

**
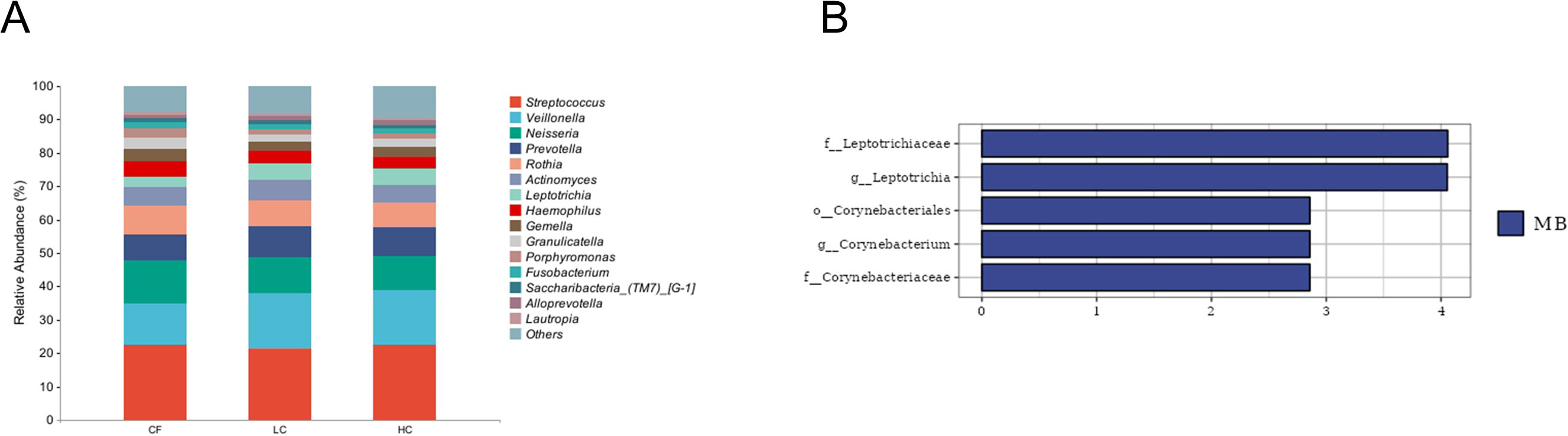
**

**Figure S1.** (A) Top 15 in abundance at the genus level among the CF, LC and HC groups. (B) LDA scores for bacterial taxa differing in abundance between the MA and MB groups


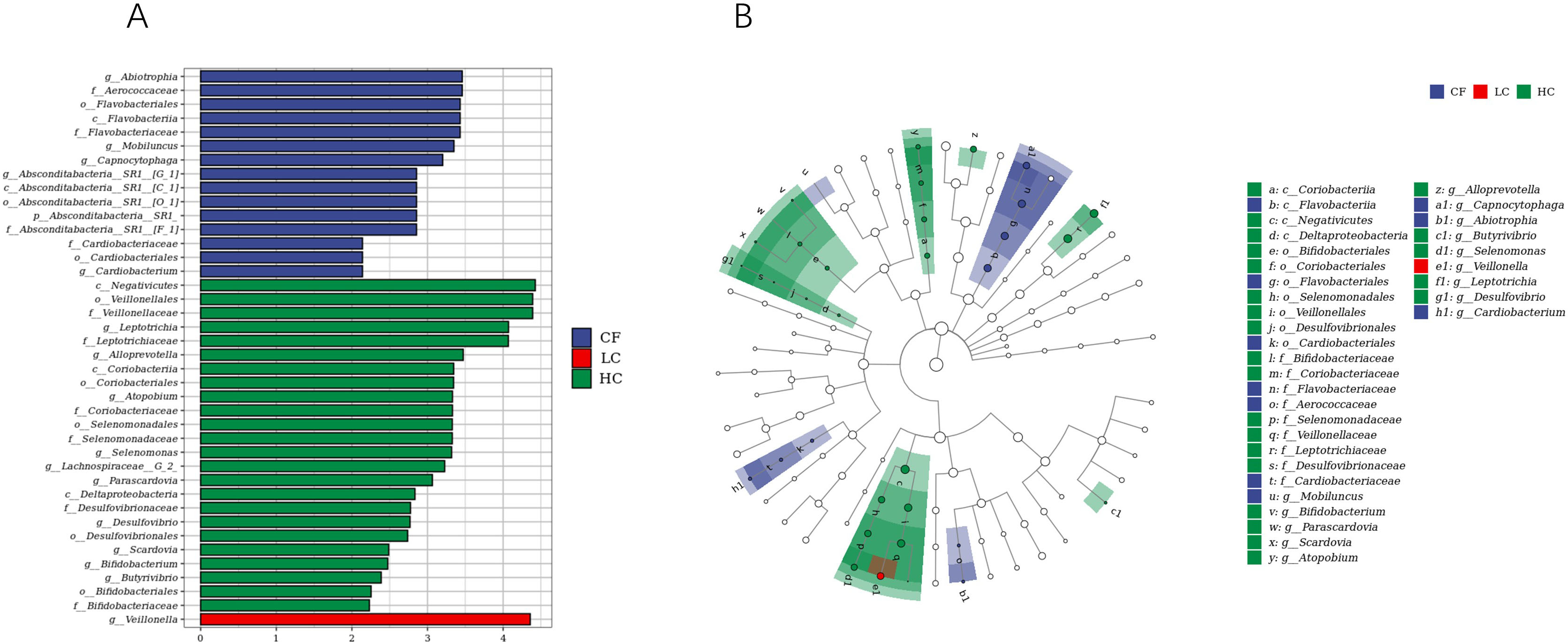


**Figure S2.** (A) LDA scores for bacterial taxa differing in abundance among different caries status groups. (B) Cladograms generated by LEfSe indicating taxonomic differences among CF, LC and HC groups.


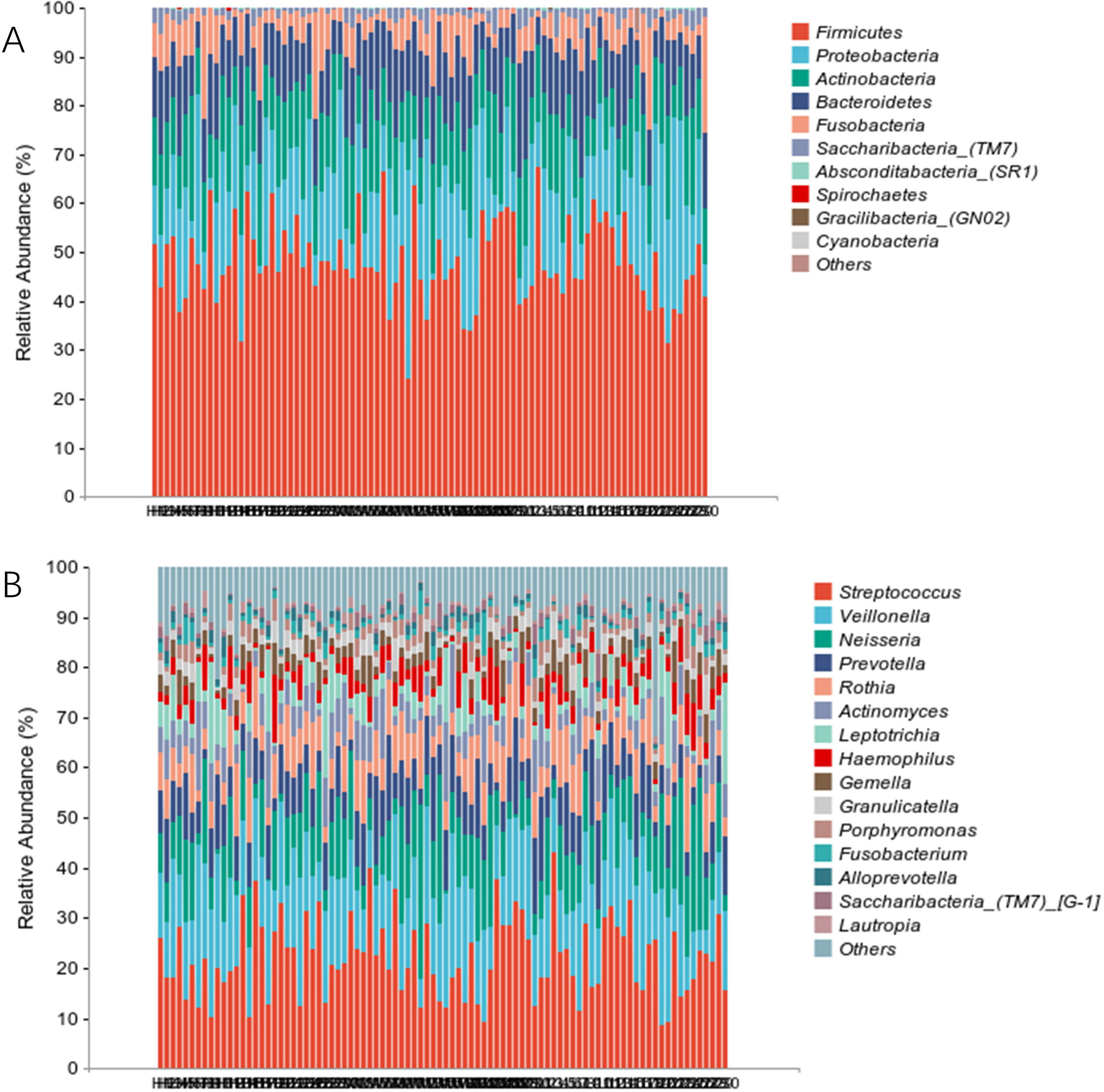


**Figure S3.** Comparison of relative abundance of 30 subjects in three CA groups (high, medium and low) in different taxonomic groups. (A) Top 10 in abundance at the phylum level, (B) Top 15 in abundance at the genus level.
